# Melatonin attenuated brain death tissue extract-induced cardiac damage by suppressing DAMP signaling

**DOI:** 10.18632/oncotarget.23180

**Published:** 2017-12-12

**Authors:** Pei-Hsun Sung, Fan-Yen Lee, Ling-Chun Lin, Kuan-Hung Chen, Hung-Sheng Lin, Pei-Lin Shao, Yi-Chen Li, Yi-Ling Chen, Kun-Chen Lin, Chun-Man Yuen, Hsueh-Wen Chang, Mel S. Lee, Hon-Kan Yip

**Affiliations:** ^1^ Division of Cardiology, Department of Internal Medicine, Kaohsiung Chang Gung Memorial Hospital and Chang Gung University College of Medicine, Kaohsiung, Taiwan; ^2^ Division of Thoracic and Cardiovascular Surgery, Department of Surgery, Kaohsiung Chang Gung Memorial Hospital and Chang Gung University College of Medicine, Kaohsiung, Taiwan; ^3^ Department of Anesthesiology, Kaohsiung Chang Gung Memorial Hospital and Chang Gung University College of Medicine, Kaohsiung, Taiwan; ^4^ Division of Neurology, Department of Internal Medicine, Kaohsiung Chang Gung Memorial Hospital and Chang Gung University College of Medicine, Kaohsiung, Taiwan; ^5^ Department of Nursing, Asia University, Taichung, Taiwan; ^6^ Division of Neurosurgery, Department of Surgery, Kaohsiung Chang Gung Memorial Hospital and Chang Gung University College of Medicine, Kaohsiung, Taiwan; ^7^ Department of Biological Sciences, National Sun Yat-Sen University, Kaohsiung, Taiwan; ^8^ Department of Orthopedics, Kaohsiung Chang Gung Memorial Hospital and Chang Gung University College of Medicine, Kaohsiung, Taiwan; ^9^ Institute for Translational Research in Biomedicine, Kaohsiung Chang Gung Memorial Hospital, Kaohsiung, Taiwan; ^10^ Center for Shockwave Medicine and Tissue Engineering, Kaohsiung Chang Gung Memorial Hospital and Chang Gung University College of Medicine, Kaohsiung, Taiwan; ^11^ Department of Medical Research, China Medical University Hospital, China Medical University, Taichung, Taiwan

**Keywords:** brain death, damage-associated molecular patterns, inflammation, remote organ, heart function

## Abstract

We tested the hypothesis that melatonin prevents brain death (BD) tissue extract (BDEX)-induced cardiac damage by suppressing inflammatory damage-associated molecular pattern (DAMP) signaling in rats. Six hours after BD induction, levels of a DAMP component (HMGB1) and inflammatory markers (TLR-2, TLR-4, MYD88, IκB, NF-κB, IL-1β, IFN-γ, TNF-α and IL-6) were higher in brain tissue from BD animals than controls. Levels of HMGB1 and inflammatory markers were higher in BDEX-treated H9C2 cardiac myoblasts than in cells treated with healthy brain tissue extract. These increases were attenuated by melatonin but re-induced with luzindole (all *P* < 0.001). Additional male rats (*n =* 30) were divided into groups 1 (negative control), 2 (healthy brain tissue extract implanted in the left ventricular myocardium [LVM]), 3 (BDEX-LVM), 4 (BDEX-LVM + melatonin), and 5 (BDEX-LVM + melatonin + luzindole). Collagen deposition/fibrosis and LVM levels of MTR2, HMGB1, inflammatory markers, oxidative stress, apoptosis, mitochondrial damage and DNA damage were highest in group 3, lowest in groups 1 and 2, and higher in group 5 than in group 4. Heart function and LVM levels of MTR1 and anti-inflammatory, mitochondrial-integrity and anti-oxidative markers exhibited a pattern opposite that of the inflammatory markers in the five groups (all *P* < 0.0001). These results indicate melatonin inhibits BDEX-induced cardiac damage by suppressing the DAMP inflammatory axis.

## INTRODUCTION

Heart transplantation is the life-saving last resort for patients with terminal heart disease [1–4]. However, two fundamental issues remain unresolved: the extremely limited number of donors, and rejection after heart transplantation. To date, there has been no effective strategy to address the first issue, while the second issue begs for the development of innovative strategies to overcome the barriers to transplant success.

Previous studies have revealed that the circulating levels of inflammatory biomarkers are markedly upregulated in patients after acute ischemic stroke [5–9]. These circulating inflammatory biomarkers increase to a much greater extent in severe ischemic stroke patients than in mild ischemic stroke patients [5–9]. Moreover, a firm association has been established between the severity of brain damage and the ensuing inflammatory reactions [5–12].

Experimental animal studies have demonstrated that after brain death (BD), vigorous inflammatory (i.e., innate) and immune (i.e., adaptive) responses frequently occur in the circulation [13] and in major organs such as the heart, kidney and liver [10–12], causing organ injury [5–16]. Moreover, damage-associated molecular patterns (DAMPs) have been identified as critical signals that trigger the inflammatory immune system in response to tissue damage [17, 18]. DAMPs include endogenous intracellular molecules released by activated or necrotic cells, as well as extracellular matrix molecules [18]. DAMPs initiate signaling cascades that activate toll-like receptors (TLRs) and MYD80, and further activate the downstream signaling of IκB, nuclear factorκB, interferon (IFN) regulatory factors, etc. [18–20]. These factors induce the expression of proinflammatory cytokines in damaged organs [18–22], and the cytokines are released into the circulation, ultimately damaging major remote organs. This sequence of events may be one of the main contributors to the risk of heart transplantation failure [23]. As heart transplant donors are always BD victims, it is important for scientists and physicians to clarify not only the underlying mechanisms of BD-induced remote cardiac damage, but also safe and effective therapeutic options to reduce such damage.

Melatonin (N-acetyl-5-methoxytryptamine), an indoleamine that is produced in the pineal gland, retina, bone marrow and bile [24], was initially identified as a neurohormonal peptide that primarily participates in the circadian regulation of physiological and neuroendocrine functions. Melatonin and its derived metabolites are also potent scavengers of free radicals and reactive oxygen and nitrogen species [25, 26], including the highly toxic hydroxyl radical (.OH), peroxynitrite anion (ONOO-) and hypochlorous acid (HOCl), which all contribute to the inflammatory response and associated tissue destruction [24]. Additionally, melatonin and its derivatives can prevent nuclear factor (NF)-κB from translocating to the nucleus and binding to DNA, thereby suppressing the upregulation of a variety of proinflammatory cytokines [24, 27] and reducing tissue/organ damage [25, 28–31]. Accordingly, while exploring the mechanism of BD-induced remote organ damage [10–16, 18–22] and the suppressive effect of melatonin on inflammation/oxidative stress [25–31], we tested the hypothesis that melatonin treatment would reduce the remote cardiac damage caused by BD-derived inflammation.

## RESULTS

### Pathophysiological findings 6 hours after the BD procedure

**Figure 1:**
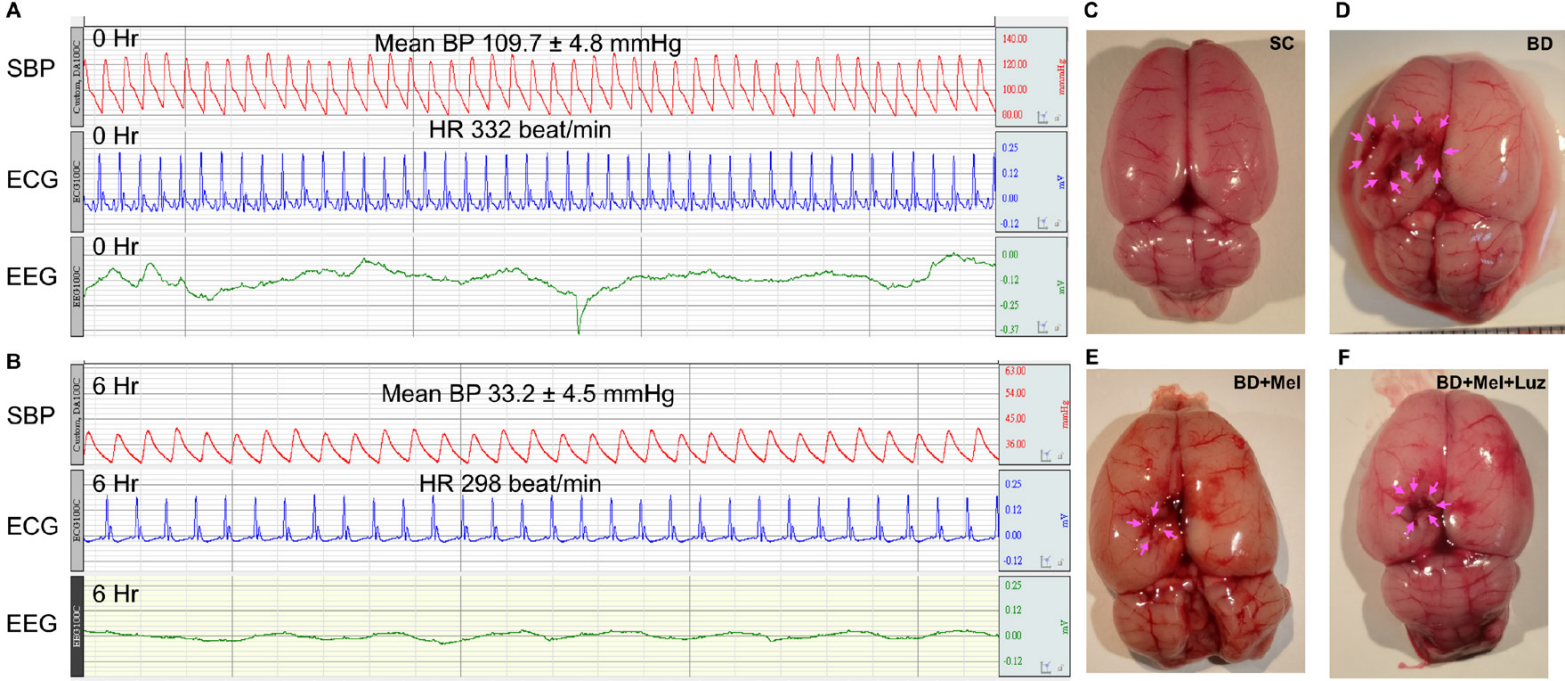
Pathophysiological findings at 6 h after the BD procedure (**A**–**B**) Illustrating the SBP (systolic blood pressure), ECG (electrocardiogram) and EEG (electroencephalogram) tracing at baseline and at 6 h after BD induction. (**C**–**F**) Illustrating the gross anatomy of brain organ in SC (C), BD (D), BD + Mel (E) and BD + Mel + Luz (F), respectively. Pink color arrows indicated the pressure applied into the brain that induced ischemic and hemorrhagic appearance. BD = brain death; Mel = melatonin; Luz = luzindole.

Figure 1 illustrates the pathophysiological findings (i.e., heart rate and blood pressure) in BD rats. During the BD period (maintained for 6 hours), the mean heart rate was about 226.5 ± 7.5 beats/min, the mean blood pressure was 33.2 ± 4.5 mmHg, and the evoked potential (eV) measured with an electroencephalogram was 0.42 at baseline and 0.039 six hours after the BD procedure, demonstrating that the experimental model of BD was successfully created (Figure 1). Additionally, as shown in the gross anatomical picture, the pressure area of the brain had a local ischemic and hemorrhagic appearance, confirming the appropriate application of pressure-induced BD in the animals (Figure 1).

### Expression of DAMP inflammatory signaling proteins in BD

**Figure 2:**
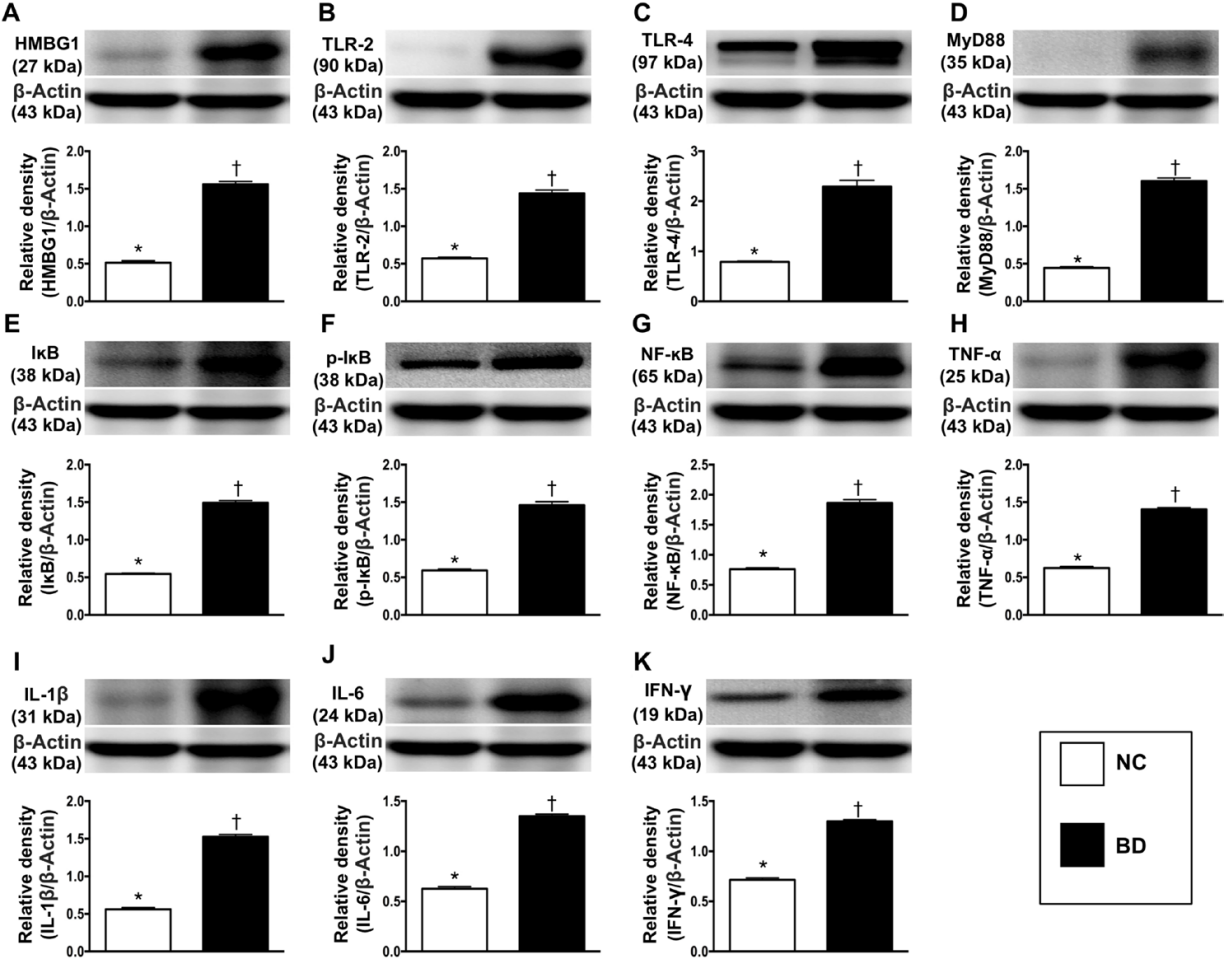
Protein expressions of DAMPs-inflammatory signaling in brain tissue (**A**) Protein expression of high mobility group box 1 (HMGB1), ^*^ vs. ^†^*p* < 0.001. (**B**) Protein expression of toll-like receptor (TLR)-2, ^*^ vs. †*p* < 0.01. (**C**) Protein expression of TLR-4, ^*^ vs. ^†^*p* < 0.01. (**D**) Protein expression of myeloid differentiation primary response gene 88 (MyD88), ^*^ vs. ^†^*p* < 0.003. (**E**) Protein expression of IκB, ^*^ vs. ^†^*p* < 0.001. (**F**) Protein expression of phosphorylated (p)-IκB, ^*^ vs. †*p* < 0.001. (**G**) Protein expression of nuclear factor (NF)-κB, ^*^ vs. ^†^*p* < 0.01. (**H**) Protein expression of tumor necrosis factor (TNF)-α, ^*^ vs. †*p* < 0.003. (**I**) Protein expression of interleukin (IL)-1ß, ^*^ vs. ^†^*p* < 0.003. (**J**) Protein expression of IL-6, ^*^ vs. †*p* < 0.002. (**K**) Protein expression of FN-γ, ^*^ vs. ^†^*p* < 0.01. *n* = 4 in each group. NC = normal control; BD = brain death. DAMPs = damage-associated molecular patterns.

The protein expression of high mobility group box 1 (HMGB1), a major DAMP component, was significantly greater in BD animals than in normal control (NC) animals, suggesting that DAMPs were released from cells (e.g., injured neurons, oligodendrocyte-like cells, glial cells) and tissues after BD (Figure 2).

To determine the putative target receptors of DAMPs, we performed Western blot analysis. The protein levels of TLR-2 and TLR-4 (two target receptors of DAMPs) were significantly higher in BD animals than in NC animals (Figure 2). The expression of MYD88 (a ligand in the TLR-dependent response that stimulates the production of proinflammatory cytokines) followed the same pattern as TLR-2 and TLR-4 in the two groups, implying that the TLR2, TLR4 and MYD88 signaling pathway might be involved in the DAMP-induced expression of inflammatory cytokines (Figure 2).

To assess the downstream signaling of proinflammatory cytokines, we once again performed Western blot analysis. The protein levels of IκB, phosphorylated (p)-IκB, NF-κB, tumor necrosis factor alpha (TNF-α), interleukin (IL)-1ß, IL-6 and IFN-γ, seven inflammatory biomarkers, exhibited the same pattern as MYD88 in BD and NC animals (Figure 2).

### The therapeutic impact of melatonin on DAMP inflammatory signaling protein levels in the context of BD

**Figure 3:**
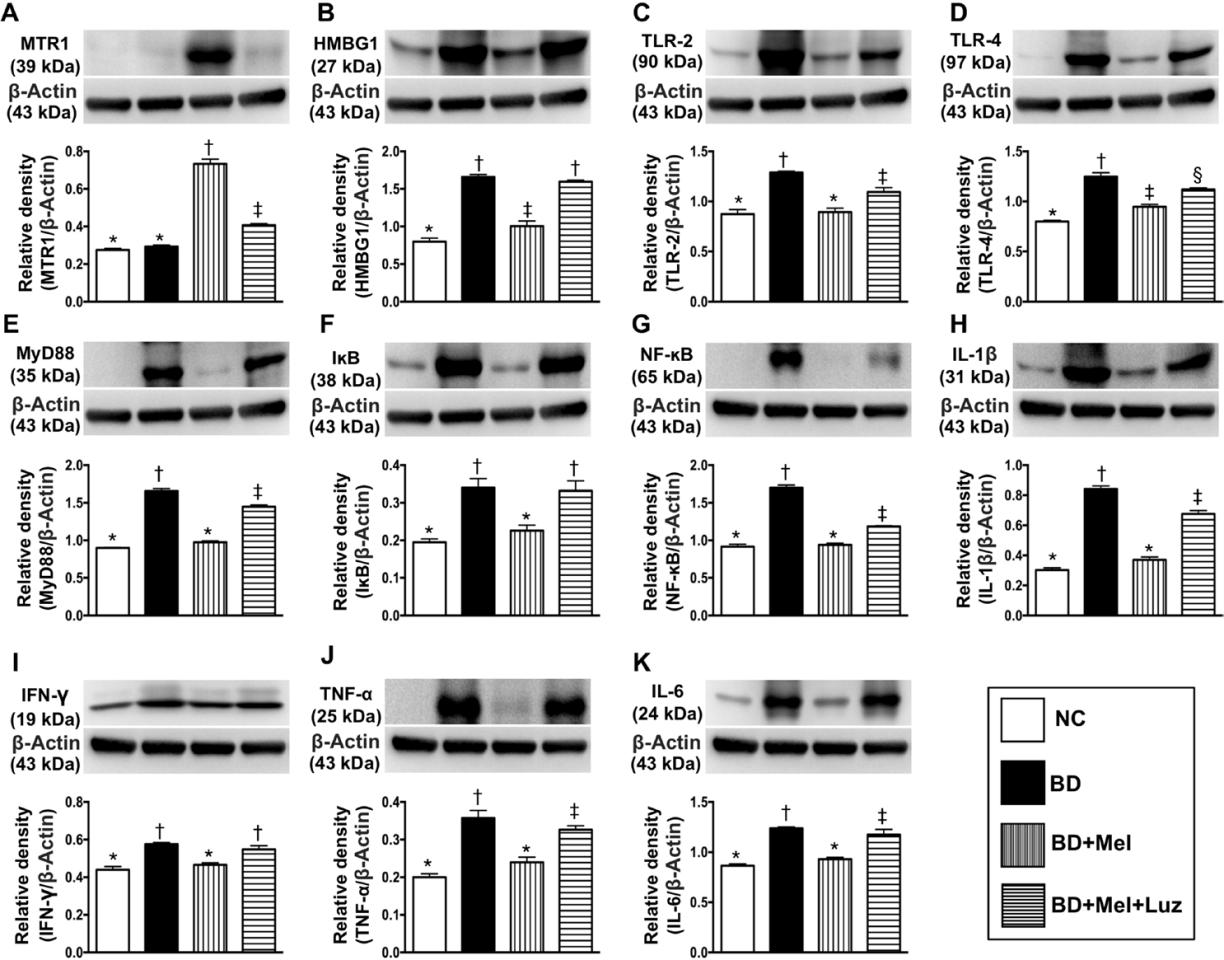
Investigated the therapeutic impact of melatonin on protein expressions of DAMPs-inflammatory signaling in setting of BD (**A**) Protein expression of melatonin receptor 1 (MTR1), ^*^ vs. other groups with different symbols (^†^, ^‡^), *p* < 0.0001. (**B**) High-mobility group box-1 (HMGB-1), ^*^ vs. other groups with different symbols (†, ‡), *p* < 0.001. (**C**) Protein expression of toll-like receptor (TLR)-2, ^*^ vs. other groups with different symbols (†, ‡), *p* < 0.001. (**D**) Protein expression of TLR-4, ^*^ vs. other groups with different symbols (^†^, ^‡^, §), *p* < 0.001. (**E**) Protein expression of myeloid differentiation primary response gene 88 (MyD88), ^*^ vs. other groups with different symbols (^†^, ^‡^), *p* < 0.001. (**F**) Protein expression of IκB, ^*^ vs. other groups with different symbols (^†^, ^‡^, ^§^), *p* < 0.001. (**G**) Protein expression of nuclear factor (NF)-κB, ^*^ vs. other groups with different symbols (^†^, ^‡^), *p* < 0.001. (**H**) Protein expression of interleukin (IL) – 1ß, ^*^ vs. other groups with different symbols (^†^, ^‡^), *p* < 0.0001. (**I**) Protein expression of interferon (IFN)-γ, ^*^ vs. †, *p* < 0.01. (**J**) Protein expression of tumor necrosis factor (TNF)-α, ^*^ vs. other groups with different symbols (†, ‡), *p* < 0.0001. (**K**) Protein expression of IL-6, ^*^ vs. other groups with different symbols (^†^, ^‡^), *p* < 0.001. All statistical analyses were performed by one-way ANOVA, followed by Bonferroni multiple comparison post hoc test (*n* = 6 for each group). Symbols (^*^, ^†^, ^‡^, ^§^) indicate significance (at 0.05 level). NC = normal control; BD = brain death; Mel = melatonin; Luz = luzindole.

To examine the effects of melatonin treatment following BD, we performed further animal studies with NC animals (healthy brains), BD animals, BD animals treated with melatonin (BD + Mel), and BD animals treated with melatonin and its antagonist, luzindole (BD + Mel + Luz). Western blot analysis was conducted to assess the expression of melatonin receptor 1 (MTR1) and DAMP inflammatory signaling proteins in brain tissue. The protein expression of MTR1, a melatonin-specific receptor mainly localized in the brain, did not differ between the NC and BD groups. However, MTR1 expression was significantly higher in the BD + Mel group than in the NC and BD groups, and this effect was notably reversed in the BD + Mel + Luz group, suggesting that MTR1 expression was enhanced by melatonin and suppressed by luzindole (Figure 3).

The protein expression of the HMGB1 was significantly higher in BD than in NC rats. While this increase was significantly reversed in the BD + Mel group, HMGB1 was upregulated again in the BD + Mel + Luz group (Figure 3). Additionally, the protein levels of TLR-2, TLR-4 and MYD88 were highest in the BD group, lowest in the NC group, and significantly lower in the BD + Mel group than in the BD + Mel + Luz group (Figure 3).

As expected, the protein levels of IκB (a kinase enzyme complex that is part of the upstream NF-κB signaling cascade) and NF-κB (a protein complex that induces DNA transcription and cytokine production) were significantly higher in BD than in NC rats, and were significantly downregulated in BD + Mel rats, but were upregulated in BD + Mel + Luz rats (Figure 3). Additionally, the protein levels of IL-1ß, IFN-γ, TNF-α and IL-6, four downstream inflammatory cytokines, displayed the same pattern as NF-κB in the BD and NC groups (Figure 3). These parameters were significantly downregulated in the BD + Mel group, and were re-upregulated in the BD + Mel + Luz group (Figure 3).

### The therapeutic impact of melatonin on DAMP inflammatory signaling proteins in rat H9C2 cells (cardiac myoblasts)

**Figure 4:**
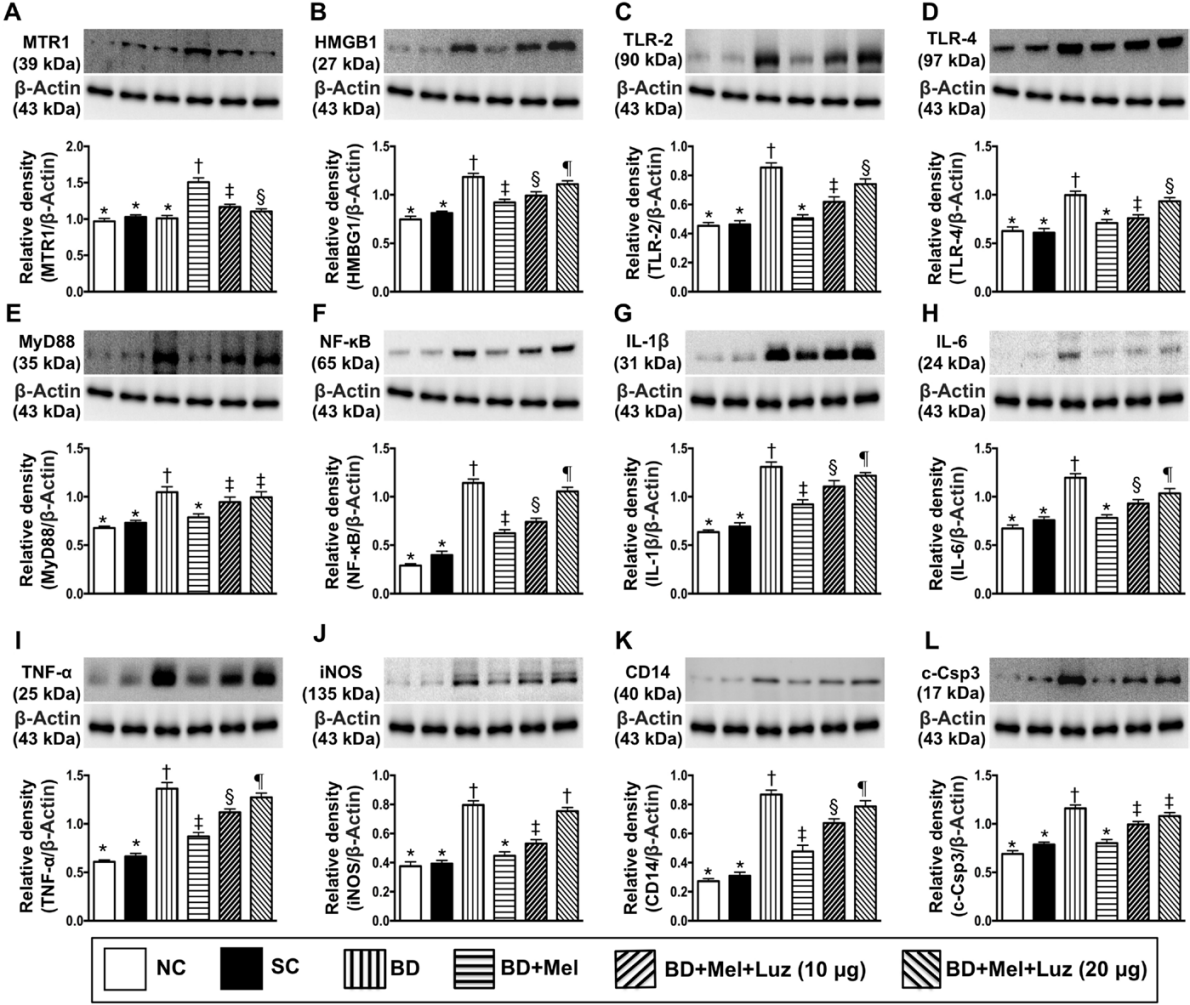
Investigated therapeutic impact on the protein expressions of DAMPs-inflammatory signaling in rat H9C2 cell line (i.e., cardiac myoblast) (**A**) Protein expressions of melatonin receptor 1 (MTR1), ^*^ vs. other groups with different symbols (^†^, ^‡^, ^§^), *p* < 0.001. (**B**) Protein expression of high mobility group box 1 (HMGB1), ^*^ vs. other groups with different symbols (^†^, ^‡^, ^§^, ^¶^), *p* < 0.001. (**C**) Protein expression of toll-like receptor (TLR)-2, ^*^ vs. other groups with different symbols (^†^, ^‡^, ^§^), *p* < 0.0001. (**D**) Protein expression of TLR-4, ^*^ vs. other groups with different symbols (^†^, ^‡^, ^§^), *p* < 0.001. (**E**) Protein expression of myeloid differentiation primary response gene 88 (MyD88), ^*^ vs. other groups with different symbols (^†^, ^‡^), *p* < 0.001. (**F**) Protein expression of nuclear factor (NF)-κB, ^*^ vs. other groups with different symbols (^†^, ^‡^, ^§^, ^¶^), *p* < 0.0001. (**G**) Protein expression of interleukin (IL)-1ß, ^*^ vs. other groups with different symbols (^†^, ^‡^, ^§^, ¶), *p* < 0.0001. (**H**) Protein expression of IL-6, ^*^ vs. other groups with different symbols (^†^, ^‡^, ^§^), *p* < 0.001. (**I**) Protein expression of tumor necrosis factor (TNF)-α, ^*^ vs. other groups with different symbols (^†^, ^‡^, ^§^, ^¶^), *p* < 0.001. (**J**) Protein expression of inducible nitric oxide synthase (iNOS), ^*^ vs. other groups with different symbols (^†^, ^‡^), *p* < 0.001. (**K**) Protein expression of CD14, ^*^ vs. other groups with different symbols (^†^, ^‡^, ^§^, ¶), *p* < 0.0001. (**L**) Protein expression of cleaved-caspase 3 (c-Casp 3), ^*^ vs. other groups with different symbols (^†^, ^‡^), *p* < 0.001. All statistical analyses were performed by one-way ANOVA, followed by Bonferroni multiple comparison post hoc test (*n* = 6). Symbols (^*^, ^†^, ^‡^, ^§^, ¶) indicate significance (at 0.05 level). NC = normal control; SC = sham control, BD_EX_ = brain death extract; Mel = melatonin; Luz = luzindole; DAMPs = damage-associated molecular patterns

We next evaluated the effects of BD on cardiac cells by co-culturing rat H9C2 cardiac myoblasts with BD-derived tissue extracts (BD_EX_), healthy brain tissue extracts (sham control, SC) or the reagent (NC). In addition, we determined the influence of melatonin and its antagonist on BD_EX_-treated H9C2 cells. The protein expression of MTR1 was highest in the BD_EX_-Mel group, lowest in the NC, SC and BD_EX_ groups (among which it did not differ significantly), and significantly lower in cells co-cultured with BD_EX_, melatonin (100 μM) and luzindole (20 μM) (BD_EX_-Mel-Luz^2^) than in cells co-cultured with BD_EX_, melatonin (100 μM) and luzindole (10 μM) (BD_EX_-Mel-Luz^1^) (Figure 4).

The protein expression of HMGB1 was significantly higher in BD_EX_ than in NC and SC cells. HMGB1 was significantly downregulated in BD_EX_-Mel cells, but was re-upregulated in BD_EX_-Mel-Luz^1^ cells and further upregulated in BD_EX_-Mel-Luz^2^ cells. However, this parameter did not differ between the NC and SC groups (Figure 4). TLR-2, TLR-4, MYD88, NF-κB, IL-1ß, IL-6, TNF-α, iNOS, CD14 and cleaved caspase 3 protein levels followed a similar pattern to HMGB1 levels among the groups (Figure 4). Our findings suggested that DAMP inflammatory signaling occurred not only *in vivo* (in BD animals), but also *in vitro* (in H9C2 cells) (Figure 4).

### Cellular infiltration and cellular biomarkers of DNA damage in the left ventricular (LV) myocardium, 5 days after BD-derived tissue implantation

**Figure 5:**
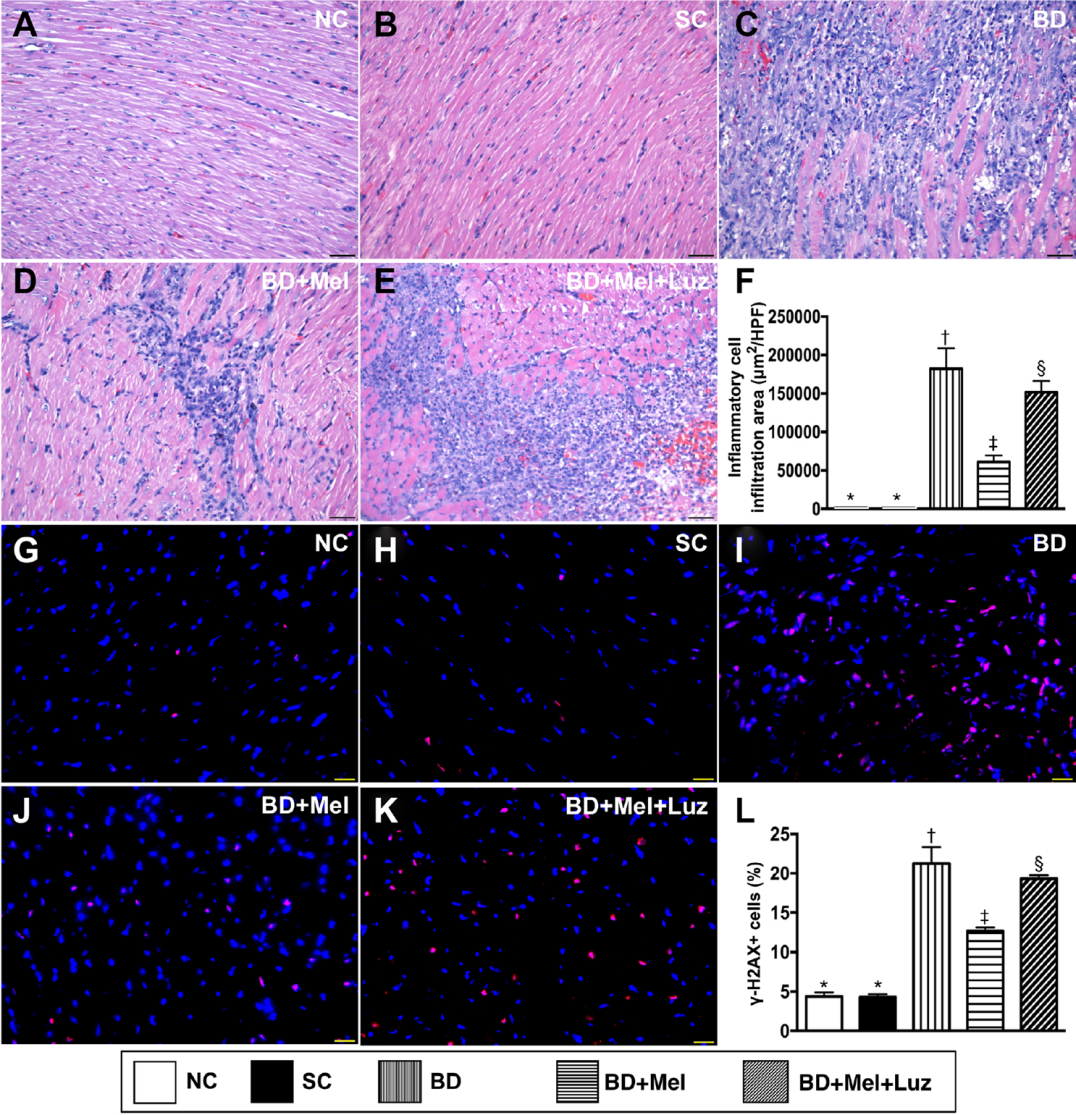
Cellular Infiltration and DNA-damaged cellular biomarker in LV myocardium by day 5 after BD-derived tissue implantation (**A**–**E**) Showing the H.&E. stain for identification of inflammatory cellular infiltration in LV myocardium (blue color). (**F**) Analytical result of cellular infiltration area, ^*^ vs. other groups with different symbols (†, ‡, §), *p* < 0.0001. (**G**–**K**) Illustrating immunofluorescent microscopic finding (400×) for identification of *γ-H2AX+ cells (pink color).* (**L**) Analytical result of number of H2AX+ cells, ^*^ vs. other groups with different symbols (^†^, ^‡^, ^§^), *p* < 0.0001. All statistical analyses were performed by one-way ANOVA, followed by Bonferroni multiple comparison post hoc test (*n* = 6). Symbols (^*^, ^†^, ^‡^) indicate significance (at 0.05 level). NC = normal control; SC = sham control; BD_EX_ = brain death extrac; Mel = melatonin; Luz = luzindole.

To assess the protective effect of melatonin against BD-induced myocardial injury, we divided adult male rats into five groups: (1) NC (only left thoracotomy), (2) SC (healthy brain tissue extracts implanted into the LV myocardium), (3) BD_EX_ (BD-derived extracts implanted into the LV myocardium), (4) BD_EX_-Mel (BD_EX_ plus melatonin treatment) and (5) BD_EX_-Mel-Luz (BD_EX_ plus melatonin and luzindole treatments). Hematoxylin and eosin staining demonstrated that the infiltration of inflammatory cells into the LV myocardium was significantly higher in the BD_EX_ group than in the NC and SC groups (which did not differ from one another) (Figure 5). This infiltration was significantly reduced in the BD_EX_-Mel group, but was re-upregulated in the BD_EX_-Mel-Luz group. The cellular expression of γ-H2AX, a DNA damage biomarker, exhibited the same pattern as hematoxylin and eosin staining in the five groups (Figure 5).

### Microscopy findings of fibrosis and condensed collagen deposition in the LV myocardium, 5 days after BD-derived tissue implantation

**Figure 6:**
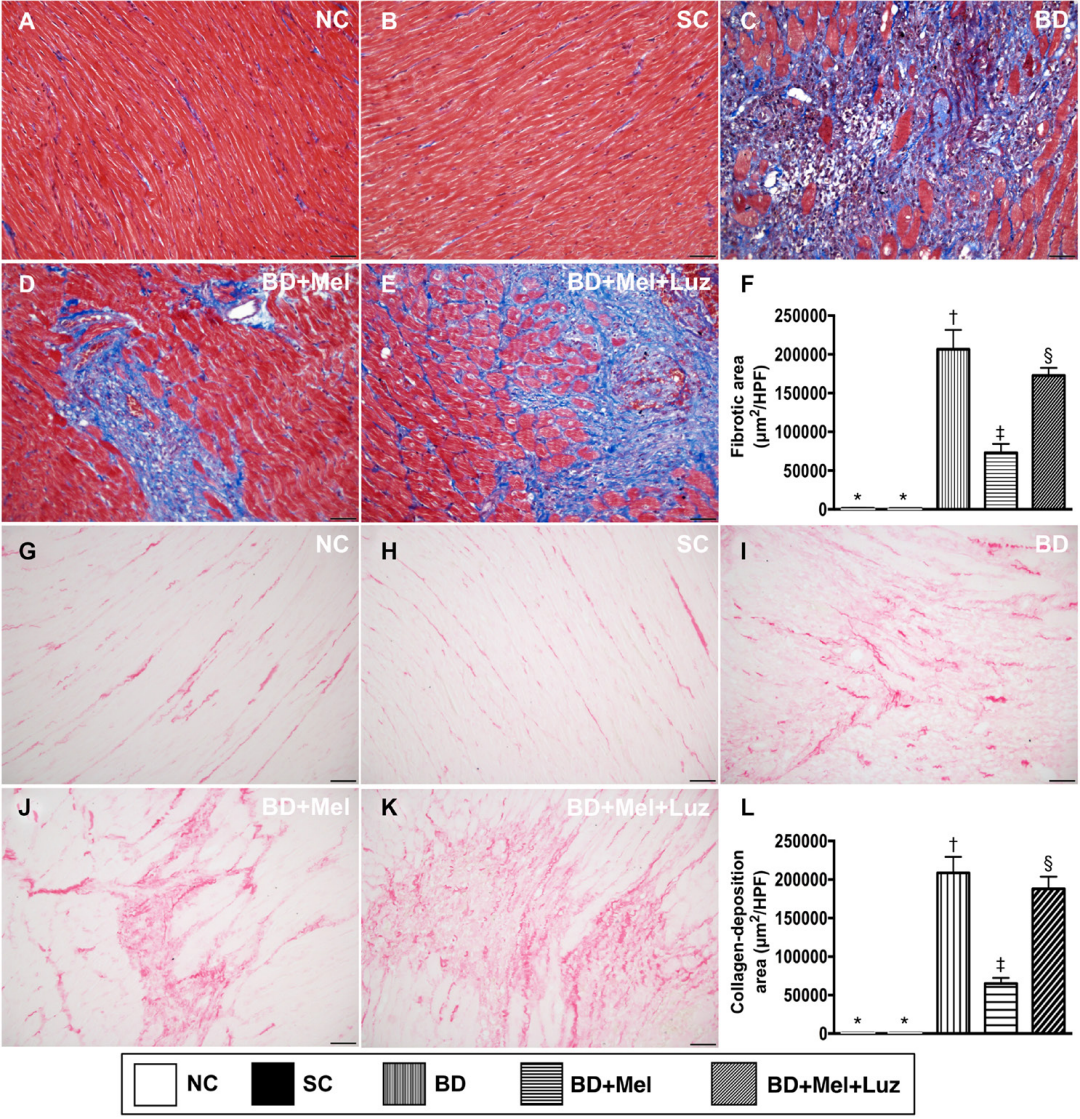
Microscopic findings of fibrotic and condensed collagen-deposition areas in LV myocardium by day 5 after BD-derived tissue implantation (**A**–**E**) Illustrating the immunohistochemical (IHC) microscopic finding (200×) for identification of fibrotic area (blue color). (**F**) Analytical result for fibrotic area, ^*^ vs. other groups with different symbols (^†^, ^‡^, ^§^), *p* < 0.0001. (**G**–**K**) Illustrating IHC microscopic finding (200×) for identification of condensed collagen-deposition area (pink color). (**L**) Analytical result of condensed collagen-deposition area, ^*^ vs. other groups with different symbols (^†^, ^‡^, ^§^), *p* < 0.0001. The scale bars in right lower corner represent 50 µm. All statistical analyses were performed by one-way ANOVA, followed by Bonferroni multiple comparison post hoc test (*n* = 6). Symbols (^*^, ^†^, ^‡^, ^§^) indicate significance (at 0.05 level). NC = normal control; SC = sham control; BD_EX_ = brain death extract; Mel = melatonin; Luz = luzindole.

We then assessed the extent of fibrosis and condensed collagen deposition in LV myocardial tissue sections from animals in the five aforementioned groups. Histochemical staining demonstrated that the fibrotic area was highest in the BD_EX_ group and lowest in the NC and SC groups, and was significantly lower in the BD_EX_-Mel group than in the BD_EX_-Mel-Luz group, but showed no difference between the NC and SC groups (Figure 6). The condensed collagen deposition area followed an identical pattern to the fibrotic area in the five groups (Figure 6).

### Inflammatory cell and immune cell infiltration in the LV myocardium, 5 days after BD-derived tissue implantation

**Figure 7:**
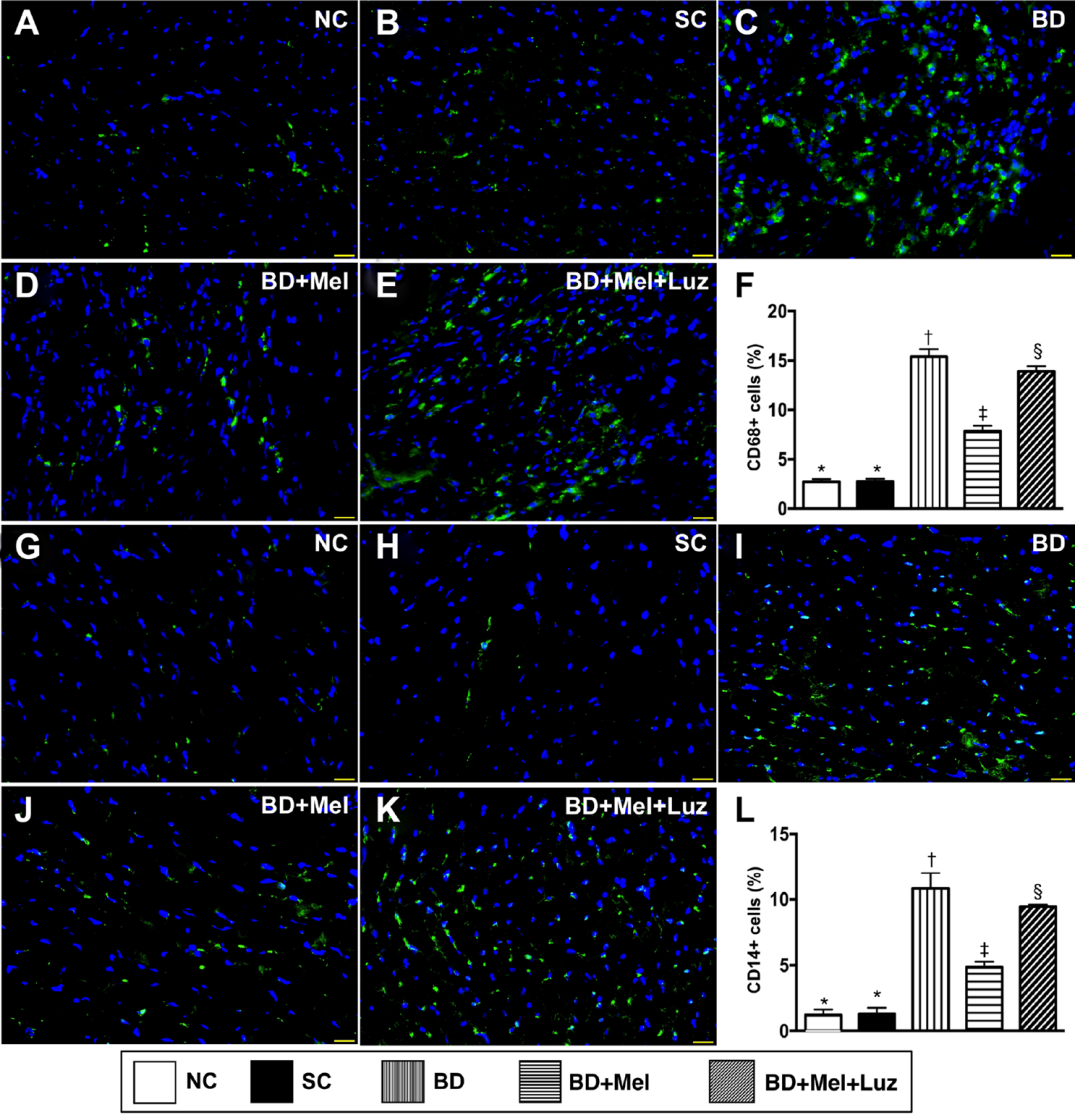
Inflammatory cell infiltration in LV myocardium by day 5 after BD-derived tissue implantation (**A**–**E**) Illustrating the immunofluorescent (IF) microscopic finding (400×) for identification of cellular expression of CD68. Nuclei were stained by DAPI (blue color). (**F**) Analytical result of number of CD68+ cells, ^*^ vs. other groups with different symbols (^†^, ^‡^, ^§^), *p* < 0.0001. (**G**–**K**) Illustrating the IF microscopic finding (400×) for identification of cellular expression of CD14. Nuclei were stained by DAPI (blue color). (**L**) Analytical result of number of CD14+ cells, ^*^ vs. other groups with different symbols (^†^, ^‡^, ^§^), *p* < 0.0001. The scale bars in right lower corner represent 20µm. All statistical analyses were performed by one-way ANOVA, followed by Bonferroni multiple comparison post hoc test (*n* = 6). Symbols (^*^, ^†^, ^‡^, ^§^) indicate significance (at 0.05 level). NC = normal control; SC = sham control; BD_EX_ = brain death extract; Mel = melatonin; Luz = luzindole.

**Figure 8:**
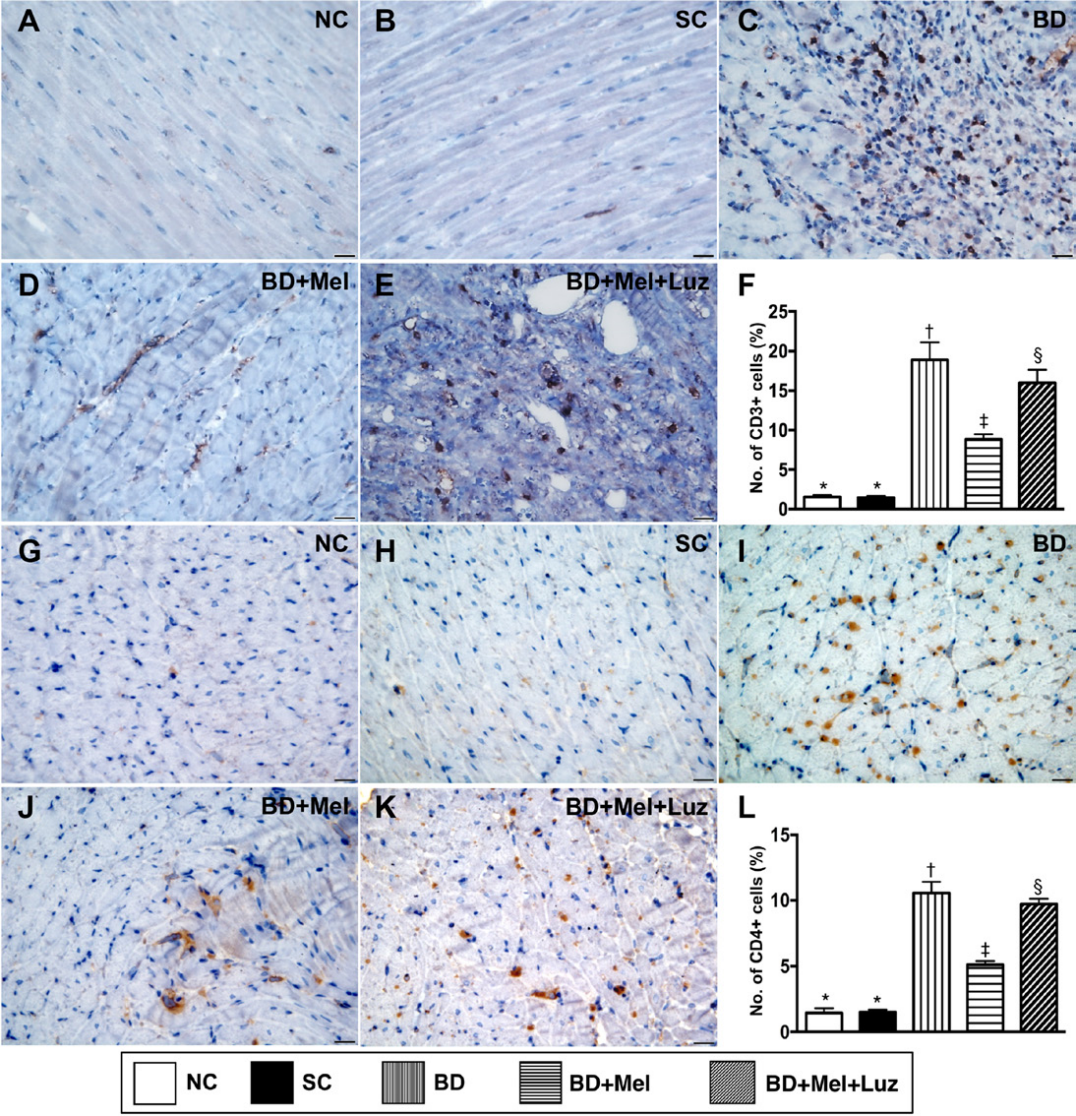
Immune cell infiltration in LV myocardium by day 5 after BD-derived tissue implantation (**A**–**E**) Demonstrating the immunohistochemical (IHC) microscopic finding (400x) for identification of cellular expression of CD3 (brown color). (**F**). Analytical result of number of CD3+ cells, ^*^ vs. other groups with different symbols (^†^, ^‡^, ^§^), *p* < 0.0001. (**G**–**K**) Demonstrating IHC microscopic finding (400×) for identification of cellular expression of CD4 (brown color). (**L**) Analytical result of number of CD4+ cells, ^*^ vs. other groups with different symbols (^†^, ^‡^, ^§^), *p* < 0.0001. The scale bars in right lower corner represent 20 µm. All statistical analyses were performed by one-way ANOVA, followed by Bonferroni multiple comparison post hoc test (*n* = 6). Symbols (^*^, ^†^, ^‡^, ^§^) indicate significance (at 0.05 level). NC = normal control; SC = sham control; BD_EX_ = brain death extract; Mel = melatonin; Luz = luzindole.

To examine inflammatory and immune cell infiltration in the LV myocardium, we performed immunohistochemistry with antibodies against CD68, CD14, CD3 and CD4. The levels of CD68 and CD14, two indicators of inflammation, were highest in the BD_EX_ group, lowest in the NC and SC groups (which did not differ significantly from one another for either protein), and significantly lower in the BD_EX_-Mel group than in the BD_EX_-Mel-Luz group (Figure 7). The expression of CD3 and CD4, two indicators of immune cells, exhibited the same pattern as CD68 in the five groups (Figure 8).

### Protein expression of inflammatory, oxidative-stress and DNA-damage biomarkers in the LV myocardium, 5 days after BD-derived tissue implantation

**Figure 9:**
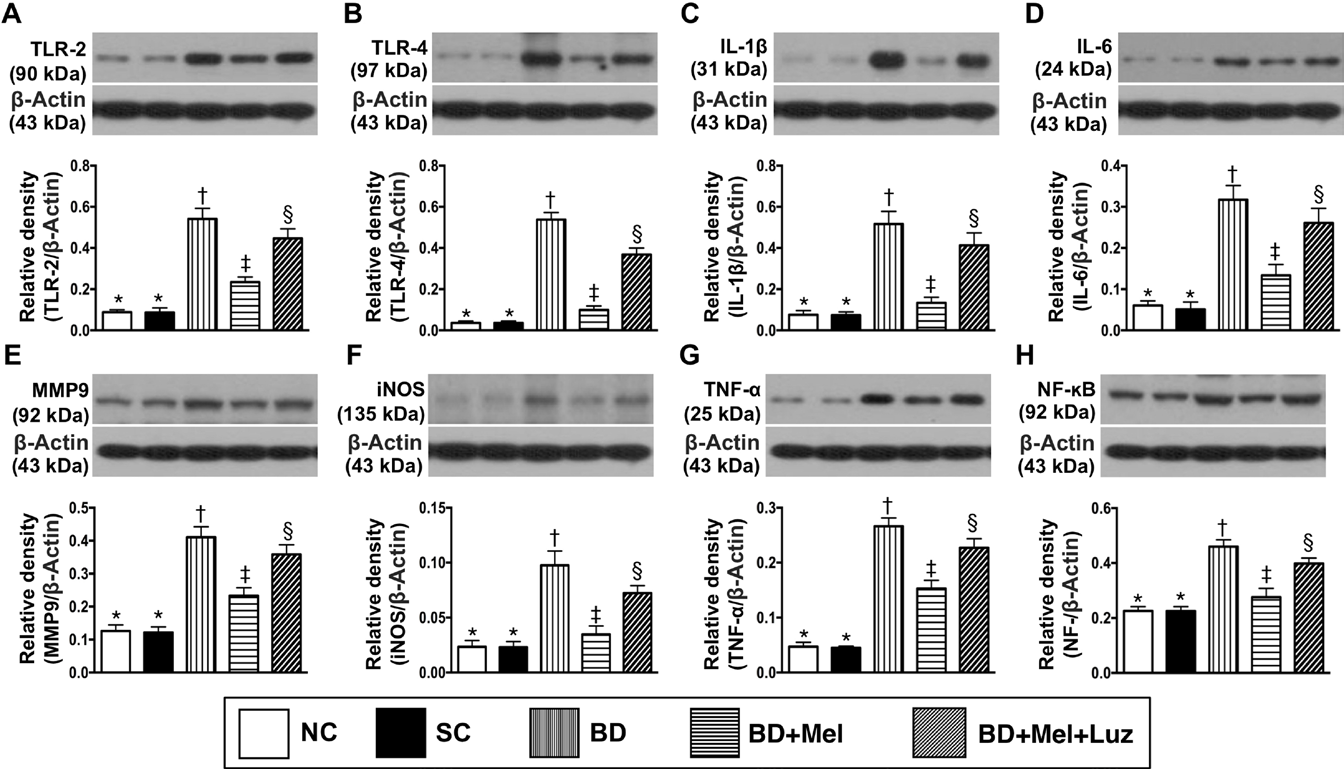
Protein expressions of inflammatory biomarkers in LV myocardium by day 5 after BD-derived tissue implantation (**A**) Protein expression of toll-like receptor (TLR)-2, ^*^ vs. other groups with different symbols (^†^, ^‡^, ^§^), *p* < 0.0001. (**B**) Protein expression of TLR-4, ^*^ vs. other groups with different symbols (^†^, ^‡^, ^§^), *p* < 0.0001. (**C**) Protein expression of interleukin (IL)-1ß, ^*^ vs. other groups with different symbols (^†^, ^‡^, ^§^), *p* < 0.0001. (**D**) Protein expression of IL-6, ^*^ vs. other groups with different symbols (†, ‡, ^§^), *p* < 0.0001. (**E**) Protein expression of matrix metalloproteinase (MMP)-9, ^*^ vs. other groups with different symbols (^†^, ^‡^, ^§^), *p* < 0.0001. (**F**) Protein expression inducible nitric oxide synthase (iNOS), ^*^ vs. other groups with different symbols (^†^, ^‡^, ^§^), *p* < 0.0001. (**G**) Protein expression of tumor necrosis factor (TNF)-α, ^*^ vs. other groups with different symbols (^†^, ^‡^, ^§^), *p* < 0.0001. (**H**) Protein expression of nuclear factor (NF) – κB, ^*^ vs. other groups with different symbols (^†^, ^‡^, ^§^), *p* < 0.0001. All statistical analyses were performed by one-way ANOVA, followed by Bonferroni multiple comparison post hoc test (*n* = 6). Symbols (^*^, ^†^, ^‡^, ^§^) indicate significance (at 0.05 level). NC = normal control; SC = sham control; BD_EX_ = brain death extract; Mel = melatonin; Luz = luzindole.

**Figure 10:**
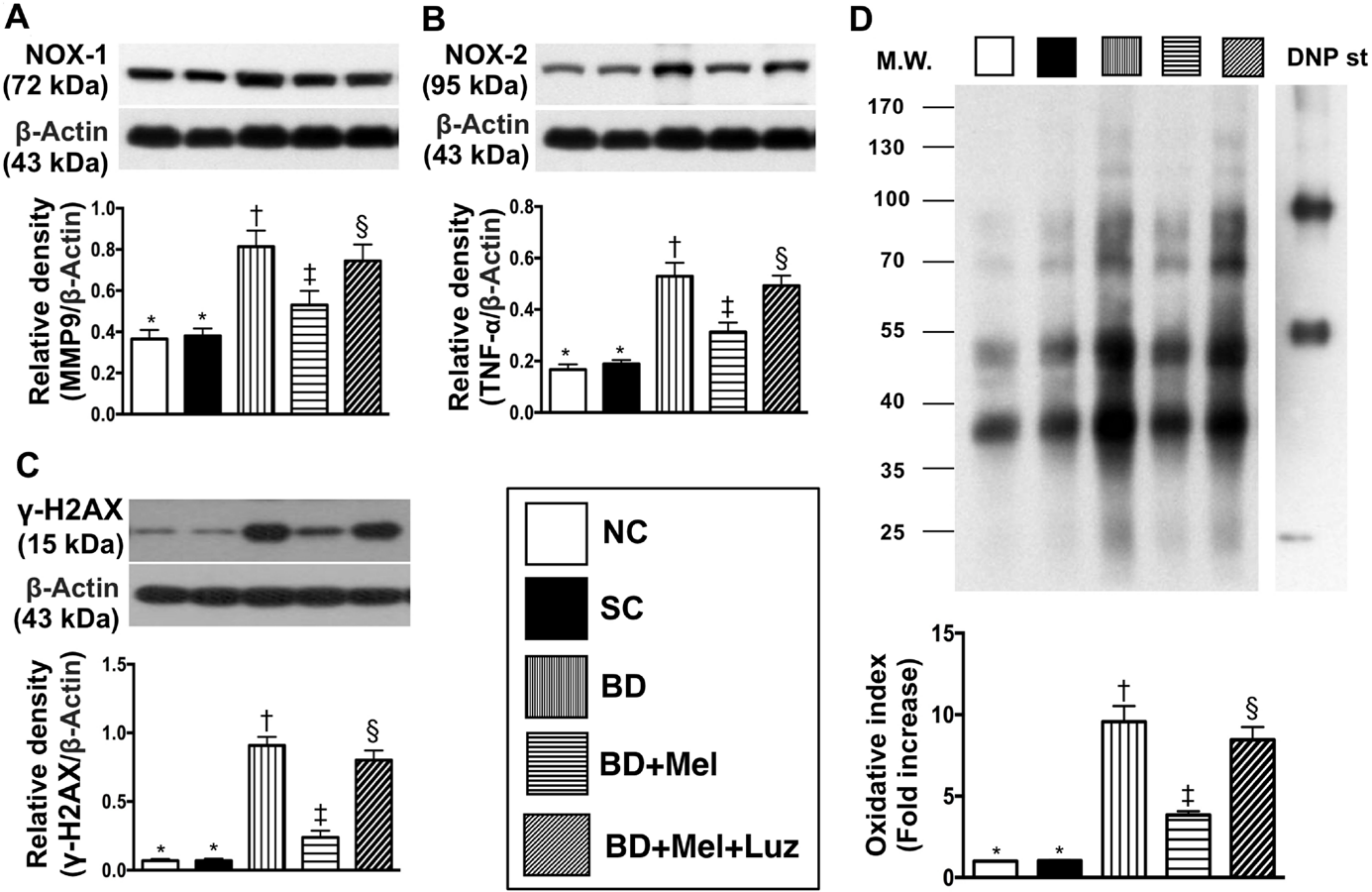
Protein expressions of oxidative-stress and DNA-damaged biomarkers in LV myocardium by day 5 after BD-derived tissue implantation (**A**) Protein expression of NOX-1, ^*^ vs. other groups with different symbols (^†^, ^‡^, ^§^), *p* < 0.001. (**B**) Protein expression of NOX-2, ^*^ vs. other groups with different symbols (^†^, ^‡^, ^§^), *p* < 0.0001. (**C**) Protein expression of *γ-H2AX,* ^*^ vs. other groups with different symbols (^†^, ^‡^, ^§^), *p* < 0.0001. (**D**) Oxidized protein expression, ^*^ vs. other groups with different symbols (^†^, ^‡^, ^§^), *p* < 0.0001. (Note: left and right lanes shown on the upper panel represent protein molecular weight marker and control oxidized molecular protein standard, respectively). M.W = molecular weight; DNP = 1–3 dinitrophenylhydrazone. All statistical analyses were performed by one-way ANOVA, followed by Bonferroni multiple comparison post hoc test (*n* = 6). Symbols (^*^, ^†^, ^‡^, ^§^) indicate significance (at 0.05 level). NC = normal control; SC = sham control; BD_EX_ = brain death extract; Mel = melatonin; Luz = luzindole.

We then performed Western blotting to measure the protein expression of eight inflammatory biomarkers in the LV myocardium. TLR-2, TLR-4, IL-1ß, IL-6, MMP-9, iNOS, TNF-α and NF-κB levels were lowest in the NC and SC groups (in which they were equivalent), highest in the BD_EX_ group, and significantly lower in the BD_EX_-Mel group than in the BD_EX_-Mel-Luz group (Figure 9). Additionally, the protein levels of NOX-1, NOX-2 and oxidized protein (three indicators of oxidative stress) and γ-H2AX (an indicator of DNA damage) followed the same pattern as the inflammatory biomarkers in the five groups (Figure 10).

### Protein expression of anti-oxidative stress, anti-inflammatory and mitochondrial-integrity biomarkers in the LV myocardium, 5 days after BD-derived tissue implantation

**Figure 11:**
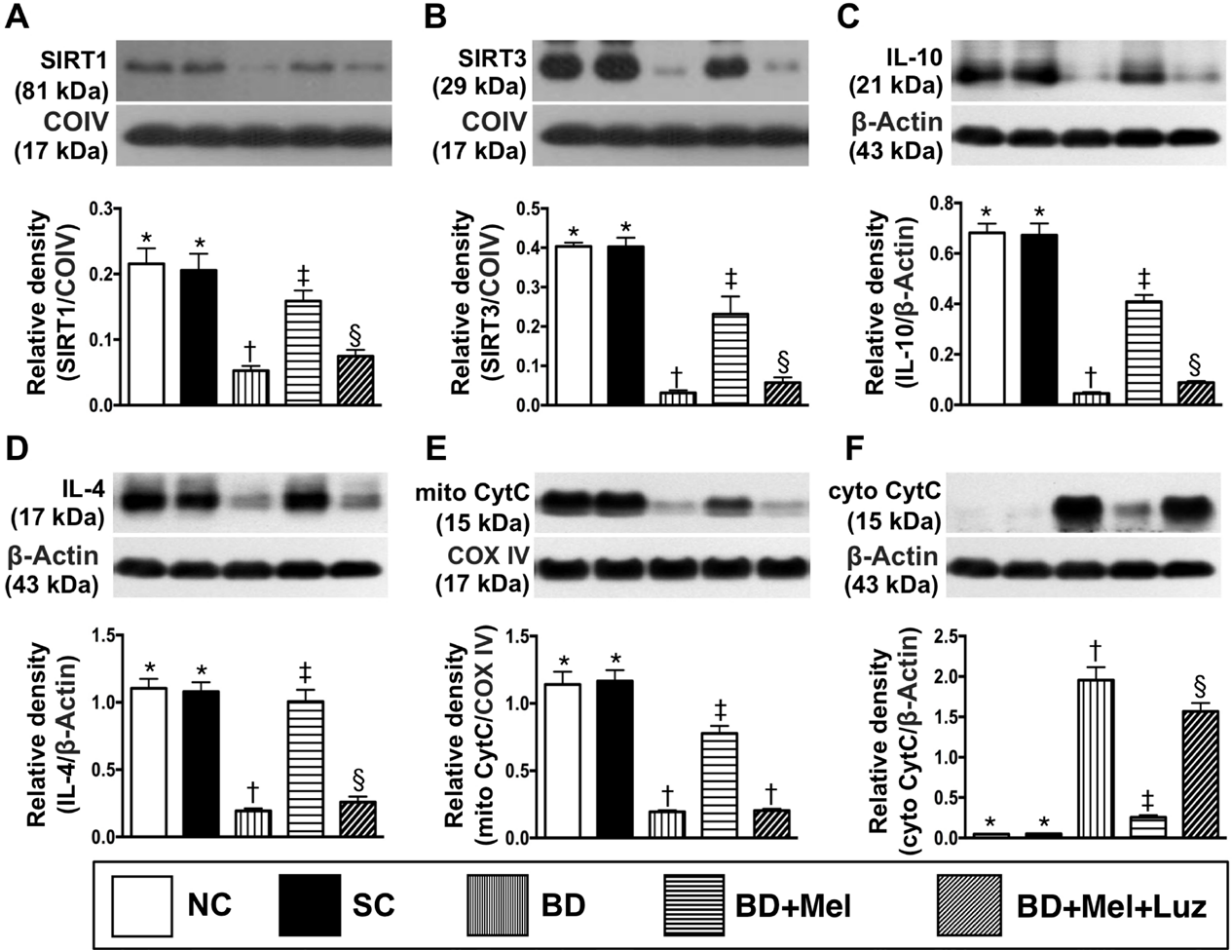
Protein expression of anti-oxidative stress, anti-inflammatory and mitochondrial integrity biomarkers in LV myocardium by day 5 after BD-derived tissue implantation (**A**) Protein expression of sirtuin 1 (SIRT1), ^*^ vs. other groups with different symbols (^†^, ^‡^, ^§^), *p* < 0.0001. (**B**) Protein expression of SIRT3, ^*^ vs. other groups with different symbols (^†^, ^‡^, ^§^), *p* < 0.0001. (**C**) The protein expression of interleukin (IL)-10, ^*^ vs. other groups with different symbols (^†^, ^‡^, ^§^), *p* < 0.0001. (**D**) Protein expression of IL-4, ^*^ vs. other groups with different symbols (^†^, ^‡^, ^§^), *p* < 0.0001. (**E**) Protein expression of mitochondrial cytochrome C (mit-Cyt C), ^*^ vs. other groups with different symbols (^†^, ^‡^), *p* < 0.0001. (**F**) Protein expression of cytosolic cytochrome C (mit-Cyt C), ^*^ vs. other groups with different symbols (^†^, ^‡^, ^§^), *p* < 0.0001. All statistical analyses were performed by one-way ANOVA, followed by Bonferroni multiple comparison post hoc test (*n* = 6). Symbols (^*^, ^†^, ^‡^, ^§^) indicate significance (at 0.05 level). NC = normal control; SC = sham control; BD_EX_ = brain death extract; Mel = melatonin; Luz = luzindole.

The LV myocardial protein levels of sirtuin (SIRT)1 and SIRT3, two anti-oxidative stress proteins, were highest in the NC and SC groups (between which they did not differ), lowest in the BD_EX_ group, and significantly higher in the BD_EX_-Mel group than in the BD_EX_-Mel-Luz group (Figure 11). The protein expression of IL-10, an anti-inflammatory biomarker, exhibited an identical pattern to the SIRTs in the five groups (Figure 11). The protein expression of IL-4, another anti-inflammatory biomarker, was significantly higher in the NC, SC and BD_EX_-Mel groups than in the BD_EX_ and BD_EX_-Mel-Luz groups, but did not differ among the first three groups or between the latter two groups (Figure 11). The protein expression of mitochondrial cytochrome C, an indicator of mitochondrial integrity, exhibited the same pattern as the SIRTs in the five groups (Figure 11). On the other hand, the protein expression of cytosolic cytochrome C, a mitochondrial damage marker, displayed the opposite pattern of mitochondrial cytochrome C (Figure 11).

### Protein levels of apoptotic markers, P53 and MTR1/MTR2 in the LV myocardium, 5 days after BD-derived tissue implantation

**Figure 12:**
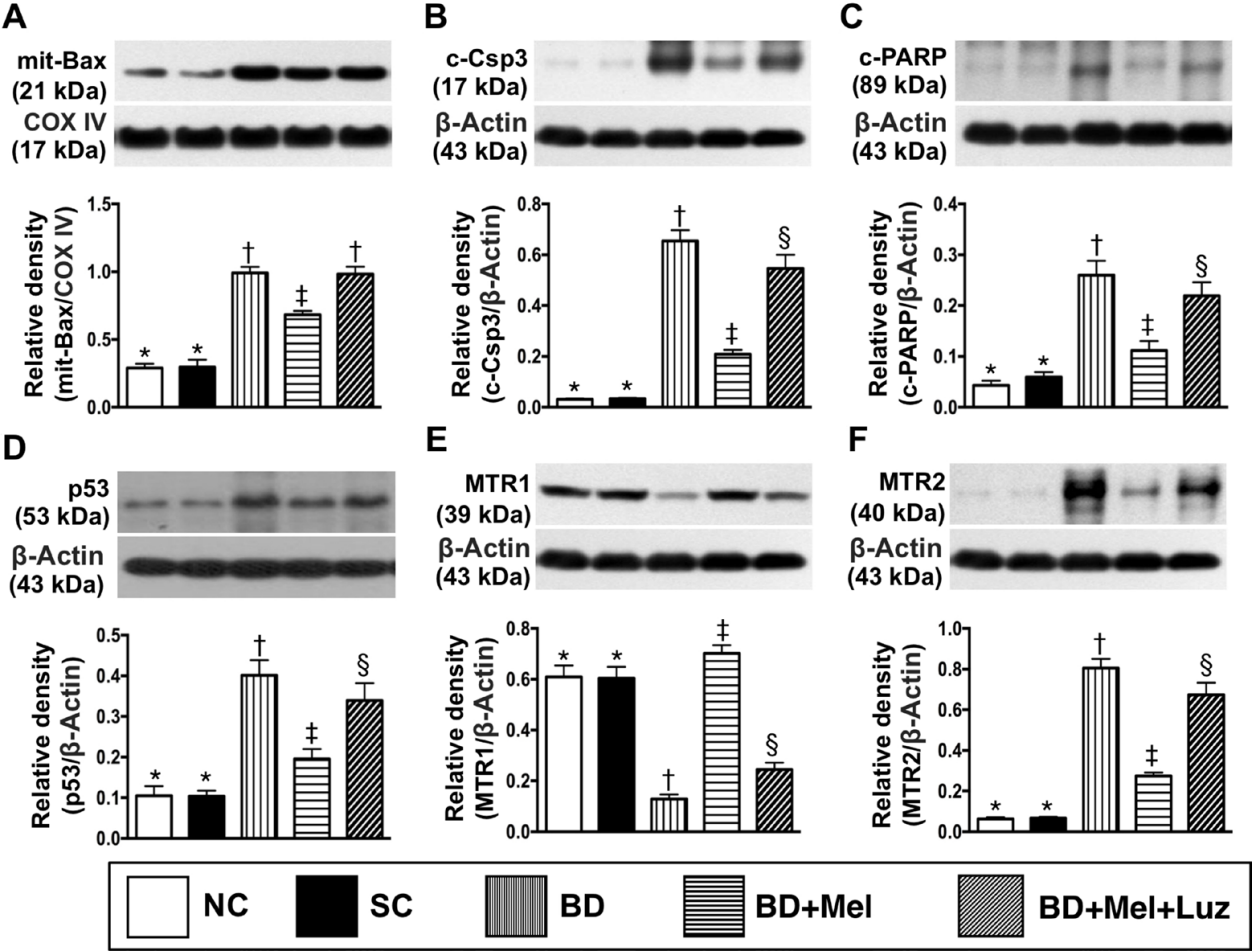
Protein expressions of apoptosis, P53 and MTR1/MTR2 in LV myocardium by day 5 after BD-derived tissue implantation (**A**) Protein expression of mitochondrial (mit)-Bax, ^*^ vs. other groups with different symbols (†, ‡), *p* < 0.0001. (**B**) Protein expression of cleaved caspase 3 (c-Casp 3), ^*^ vs. other groups with different symbols (^†^, ^‡^, ^§^), *p* < 0.0001. (**C**) Protein expression of cleaved poly (ADP-ribose) polymerase (c-PARP), ^*^ vs. other groups with different symbols (^†^, ^‡^, ^§^), *p* < 0.0001. (**D**) The protein expression of p53, ^*^ vs. other groups with different symbols (^†^, ^‡^, ^§^), *p* < 0.0001. (**E**) Protein expression of melatonin receptor (MTR)1, ^*^ vs. other groups with different symbols (^†^, ^‡^, ^§^), *p* < 0.0001. (**F**) Protein expression of MTR2, ^*^ vs. other groups with different symbols (^†^, ^‡^, ^§^), *p* < 0.0001. All statistical analyses were performed by one-way ANOVA, followed by Bonferroni multiple comparison post hoc test (*n* = 6). Symbols (^*^, ^†^, ^‡^, ^§^) indicate significance (at 0.05 level). NC normal control; SC = sham control; BD_EX_ = brain death extract; Mel = melatonin; Luz = luzindole.

The protein levels of mitochondrial BAX, cleaved caspase 3 and cleaved poly (ADP-ribose) polymerase (PARP), three indicators of apoptosis, were lowest in the NC and SC groups (between which they did not differ), highest in the BD_EX_ group, and significantly lower in the BD_EX_-Mel group than in the BD_EX_-Mel-Luz group (Figure 12). The protein expression of P53, a key inducer of cell cycle arrest to allow either cellular repair/survival or apoptosis, exhibited an identical pattern to the apoptotic markers in the five groups (Figure 12). The protein expression of MTR2 followed the same pattern as the apoptotic markers, whereas the protein expression of MTR1 displayed the opposite pattern in the five groups (Figure 12).

### Transthoracic echocardiograph findings at baseline and 5 days after BD-derived tissue implantation

We also performed transthoracic echocardiography on the five groups of animals on days 0 and 5 after BD-derived tissue implantation. On day 0, the LV end-systolic diameter (LVESd), LV end-diastolic diameter (LVEDd) and LV ejection fraction (LVEF) did not differ among the five groups (Table 1). However, by day 5 after BD-derived tissue implantation into the LV myocardium, the LVESd was significantly higher in the BD_EX_ and BD_EX_-Mel-Luz groups than in the NC, SC and BD_EX_-Mel groups, and was significantly higher in the BD_EX_-Mel group than in the NC and SC groups, but did not differ between the BD_EX_ and BD_EX_-Mel-Luz groups or between the NC and SC groups. The LVEF exhibited the opposite pattern of the LVESd among the five groups (Table 1). However, the LVEDd did not differ among the five groups (Table 1).

**Table 1:** Transthoracic echocardiographic findings at baseline and by day 5 after brain death induction

Variables	Group 1 (*n =* 6)	Group 2 (*n =* 6)	Group 3 (*n =* 6)	Group 4 (*n =* 6)	Group 5 (*n =* 6)	*P* value^*^
Day 0						
LVEDd (mm)	8.25 ± 0.27	8.43 ± 0.54	8.32 ± 0.72	8.30 ± 0.83	8.18 ± 0.59	0.667
LVESd (mm)	4.64 ± 0.18	4.77 ± 0.34	4.70 ± 0.44	4.63 ± 0.23	4.64 ± 0.32	0.874
LVEF (%)	72.64 ± 0.26	72.62 ± 0.94	72.83 ± 1.02	73.66 ± 0.61	72.58 ± 0.43	0.743
Day 5						
LVEDd (mm)	8.22 ± 0.20	8.48 ± 0.27	8.22 ± 0.62	8.34 ± 0.54	8.31 ± 0.32	0.324
LVESd (mm)	4.67 ± 0.24^a^	4.90 ± 0.30^a^	5.67 ± 0.53^b^	5.21 ± 0.24^c^	5.78 ± 0.55^b^	< 0.0001
LVEF (%)	72.05 ± 0.45^a^	71.20 ± 1.19^a^	56.26 ± 1.73^b^	64.83 ± 3.28^c^	55.33 ± 1.45^b^	< 0.0001

Data are expressed as mean ± SD. Group 1 = normal control (NC) (only left thoracotomy); Group 2 = sham control (SC) [healthy BTEX (60 μL) implanted into LV myocardium]; Group 3 = BDEX [i.e., BD-derived extraction (60 μL) implanted into LV myocardium]; Group 4 = BDEX + Mel; Group 5 = BDEX + Mel + Luz. BTEX = brain tissue extraction; BDEX = brain-death tissue extraction; Mel = melatonin; Luz = luzindole; LVEDd = left ventricular end-diastolic dimension; LVESd = left ventricular end-systolic dimension; LVEF = left ventricular ejection fraction. ^*^Letters (a, b, c) indicate significant difference (at 0.05 level) by Tukey multiple comparison procedure.

## DISCUSSION

This study investigated the remote cardiac damage induced by BD_EX_, and the protective effect of melatonin on LV function. The results yielded several striking implications. First, BD_EX_ activated DAMP inflammatory signaling, which in turn damaged the LV myocardium and impaired LV function, mainly by upregulating inflammatory (i.e., innate) and immune (i.e., adaptive) responses. Second, DAMP inflammatory signaling was only activated in BD rather than in healthy brains, indicating that the DAMPs released from BD parenchyma are crucial for *in situ* (in the BD area) and remote tissue/organ (in the LV myocardium/heart) damage. Third, melatonin therapy effectively suppressed DAMP inflammatory signaling and preserved LV function, suggesting that melatonin has potential as an accessory therapy to prevent immune rejection in patients who have undergone organ transplantation.

In our previous clinical observational studies, we found that the severity of ischemic stroke was associated with the incidences of advanced heart failure, myocardial infarction and in-hospital death, and inversely associated with LV function [5, 32]. Consistently, other experimental and clinical observation studies, including those that have investigated the correlation between BD and heart function [33–36], have had similar results [5, 32]. The most important finding in the present study was that LV function was substantially lower and LV chamber size (i.e., LV remodeling) was remarkably greater in BD_EX_ animals (with BD-derived tissue extracts implanted into the LV myocardium) than in NC and SC animals (with normal brain tissue implanted into the LV myocardium). Therefore, in addition to supporting the findings of previous studies [5, 32–36], our results indicated that only BD-derived components, but not healthy brain-derived materials, were harmful to the LV myocardium. LV function was remarkably preserved and LV remodeling was substantially suppressed in BD_EX_ animals treated with melatonin, suggesting that melatonin may be applied clinically to prevent heart-transplant rejection.

A principal finding in the present study was that the fibrotic and condensed collagen deposition areas (pathological findings) as well as DNA/mitochondrial-damage and apoptotic biomarker levels (molecular/cellular parameters) were substantially greater in BD_EX_ animals than in NC and SC animals, and were significantly reduced in BD_EX_ animals upon melatonin treatment. These findings could, at least in part, explain why LV function deteriorated and LV remodeling increased in BD_EX_ animals, and why melatonin treatment reversed these changes in BD_EX_ animals.

Intriguingly, a previous clinical observational study revealed that more than 25% LV dysfunction was present in BD patients, even at an early stage of BD [37]. Additionally, in a recent experimental study, we found that BD induced remote organ damage and acute heart-transplant rejection [38]. However, the mechanisms of BD-induced remote cardiac damage, LV dysfunction and heart-transplant rejection were not fully investigated in those studies [37, 38].

To clarify the mechanism of BD-induced LV dysfunction, we performed both *in vivo* (BD animal model) and *in vitro* (H9C2 cell line) experimental studies. We found that the protein levels of HMGB1 (a major DAMP component) and its target receptors (TLR-2 and TLR-4) were remarkably higher in BD_EX_ animals and BD_EX_-treated H9C2 cells than in their respective control groups. Additionally, the protein expression of MYD88 (a ligand in the TLR-dependent response that stimulates proinflammatory cytokine production), IκB (a kinase enzyme complex that is part of the upstream NF-κB signaling cascade) and NF-κB (a protein complex that induces DNA transcription and cytokine production) were greater in BD_EX_ animals and BD_EX_-treated H9C2 cells than in the control groups. Furthermore, in terms of downstream signal transduction, proinflammatory cytokine protein levels exhibited an identical pattern to NF-κB expression (an upstream signal of cytokine production).

**Figure 13:**
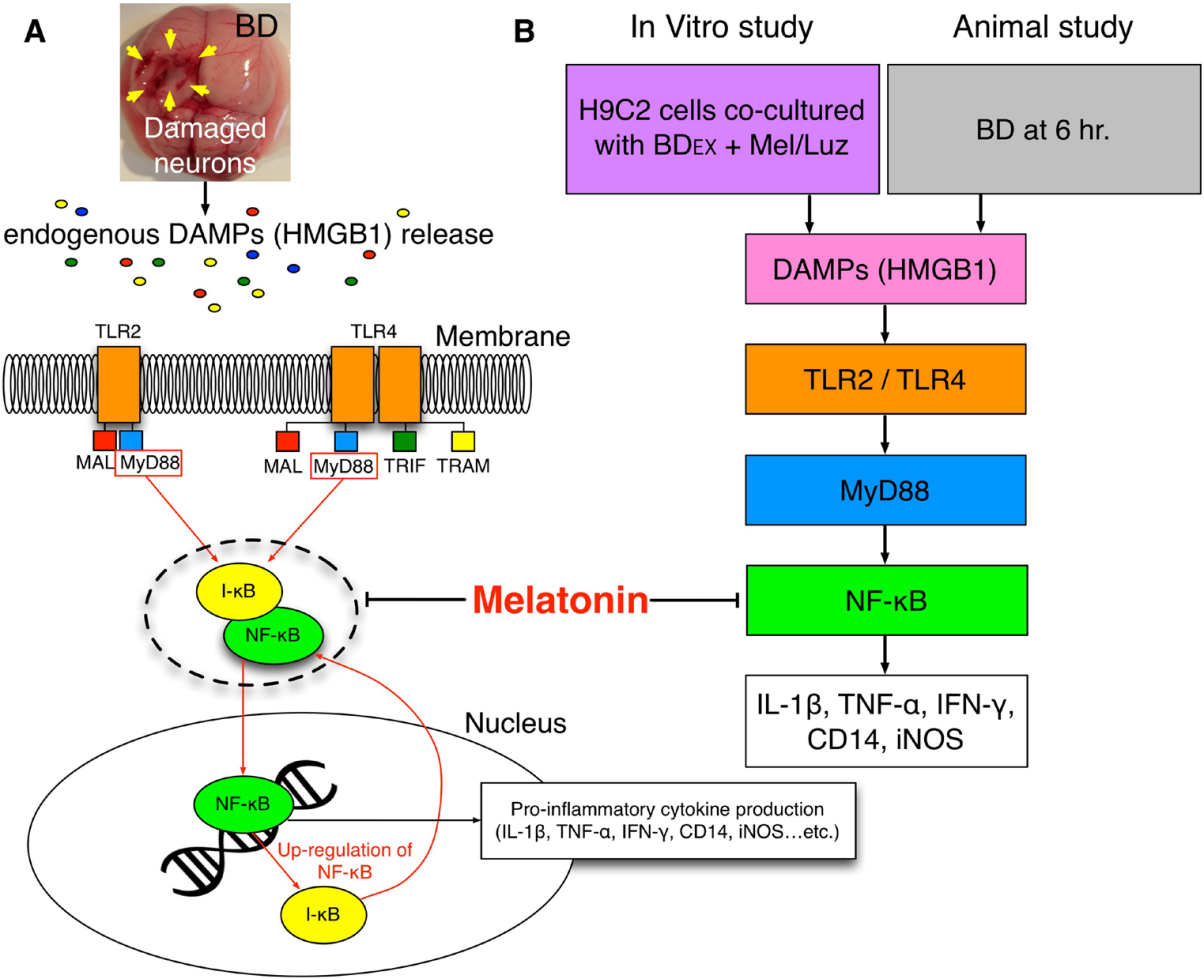
Proposed underlying mechanism of BD-induced remote cardiac organ damage through DAMPs-inflammatory signaling pathway (**A**–**B**) The propose mechanism of BD-induced release of endogenous DAMPs that elicit the inflammatory cytokines is based on the results of present study in both *in vitro* and animal studies. BD = brain death; DAMPs = damage-associated molecular patterns; HMGB = high mobility group box 1; BD_EX_ = brain death extraction; TLR = toll-like receptor; IL = interleukin; TNF-α = tumor necrosis factor alpha; IFN-γ = interferon gamma; iNOS = inducible nitric oxide synthase; NF-κB = nuclear factor-κB;

Our animal model study (rat LV myocardium implanted with BD_EX_, with and without melatonin treatment) revealed that the levels of HMGB1 and inflammatory, oxidative-stress and immune (CD3 and CD4) biomarkers were significantly higher in the BD_EX_ group than in the NC and SC groups. Our findings, therefore, have elucidated a potential mechanism (Figure 13) of DAMP inflammatory signaling and the immune response following BD-induced remote cardiac damage. In a previous review article, a similar DAMP inflammatory signaling pathway was proposed [17]. In addition to being supported by this previous report [17], our findings explain the LV dysfunction of BD patients, even in the early stage of BD [37]. Importantly, by using melatonin and luzindole (an antagonist of melatonin), we verified, in a step-by-step manner, the therapeutic potential of melatonin to inhibit the cellular/molecular and pathological perturbations in the LV myocardium, inhibit LV remodeling and improve LV function in BD_EX_ animals, mainly by altering DAMP inflammatory signaling.

A previous study found that the melatonin receptor subtypes MTR1 and MTR2 were present in the human cardiovascular system [39]. In the present study, we found that these two types of melatonin receptors were present in the rat myocardium. MTR1 expression was significantly lower in BD_EX_ animals than in NC and SC animals, but was significantly upregulated in BD_EX_ animals treated with melatonin. Surprisingly, MTR2 expression in the LV myocardium exhibited the opposite pattern of MTR1 expression in BD_EX_ animals. We remain uncertain about the reason for the discrepant expression of these two types of melatonin receptors in the LV myocardium. Perhaps a mutual effect between MTR1 and MTR2 is intrinsically present in the LV myocardium in response to ischemic stimulation.

This study has limitations. First, although the short-term effects of melatonin treatment (inhibiting DAMP inflammatory signaling, preventing LV remodeling and preserving LV function) were appealing, this study did not reveal the long-term effects of melatonin, as the study period was 5 days. Second, although we provided an attractive mechanism for BD-induced remote cardiac damage based on our extensive work (Figure 13), this mechanism may not be sufficient to explain BD-induced remote organ damage.

In conclusion, the results of the present study demonstrated that BD induced remote cardiac injury mainly by activating DAMP inflammatory signaling, while melatonin treatment significantly suppressed this pathway.

## MATERIALS AND METHODS

### Ethics statement

All animal experimental procedures were approved by the Institutional Animal Care and Use Committee at Kaohsiung Chang Gung Memorial Hospital (Affidavit of Approval of Animal Use Protocol No. 2017020801) and performed in accordance with the Guide for the Care and Use of Laboratory Animals, 8^th^ edition (NIH publication No. 85-23, National Academy Press, Washington, DC, USA, revised 2011). Animals were housed in an Association for Assessment and Accreditation of Laboratory Animal Care International-approved animal facility in our hospital, with controlled temperature and light cycles (24°C and 12/12 light/dark cycle).

### Induction of brain death in the animal model

For the induction of brain death, rats in each group were anesthetized by inhalation of 2.0% isoflurane on a warming pad at 37°C, and were placed in the prone position as described [38] to mimic the clinical scenario in which a sudden increase in intracranial pressure (as in a severe traumatic intra-cranial hemorrhage) results in brain-stem failure. An incision was made on the skin covering the skull, the epicranial muscles and periosteum were dissected, and a 4F angioplasty balloon catheter was carefully introduced into the supradural space inside the cranial cavity of the rat through a hole in the skull (made with a dental drill) at the paramedian space near the frontal transverse sinus. Each animal was endotracheally intubated with positive-pressure ventilation (180 mL/min) with room air through a small animal ventilator (SAR-830/A, CWE Inc., USA) at a ventilation rate of about 60/min. Then, 0.5 mL of distilled water was injected through the catheter into the supradurally implanted balloon to induce BD. The heart rate and blood pressure were continuously monitored during the whole procedure.

### Criteria for identifying BD in animals and harvesting BD-derived brain specimens

BD manifested as an immediate and significant reduction in the systemic arterial blood pressure and in the power density of low-frequency components of the systemic arterial blood pressure, and met the following criteria:

1. Cessation of spontaneous respiration (complete dependence on mechanical ventilation)

2. Irreversible deep coma (lack of response and reflex to pain elicited by pinching the foot with forceps)

3. Fixed and dilated pupils without a light reflex, regardless of the light intensity

The sum of the power density during the period of the low-frequency (0.25–0.8 Hz in rat) components in the blood-pressure and heart-rate spectra were computerized, along with the mean blood pressure and heart rate [40–42].

An additional six animals were used for the BD procedure. Animals were euthanized 6 hours after BD induction. The brain was removed from each animal, and brain tissues were harvested.

### Cell co-culture with brain extract

The rat cardiac myoblast cell line H9C2 was purchased from the Food Industry Research and Development Institute (Hsinchu 300, Taiwan, ROC). H9C2 cells were cultured in Dulbecco’s Modified Eagle’s Medium (Gibco/BRL, Grand Island, NY, USA) containing 10% fetal bovine serum (Hyclone, New Zealand) and 1% penicillin/streptomycin (Gibco, USA). H9C2 cells were treated with brain tissue extracts at a concentration of 1.0 μg/mL for 24 hours. The total cell lysates were harvested for Western blotting.

### Preparation of brain tissue extracts for injection into the LV myocardium

Brain tissue (100 mg) was extracted, homogenized in 1.0 mL PBS containing a protease inhibitor (539134, Millipore), and vortexed. The crude extracts were pelleted by centrifugation (1380 RCF for 5 minutes at 4°C), and the supernatants were frozen at –80°C for subsequent experiments. Protein assay dye (Bio-Rad, Hercules, California, USA) was used to quantify the brain extract concentration.

### Melatonin and luzindole treatment

Melatonin (M5250) and luzindole (L2407) were purchased from Sigma Chemical Co., USA. Melatonin was dissolved in ethanol, and luzindole was dissolved in dimethyl sulfoxide, immediately prior to utilization. The dosage of melatonin was based on our previous reports with minimal modification [29–31]. Melatonin (50 mg/kg) was administrated intraperitoneally to the BD animals 3 hours before and 3 hours after the BD procedure. To determine the antagonistic effect of luzindole against melatonin, we administered luzindole (100 μg) via the intra-carotid artery 4 hours before and 3 hours after the BD procedure.

### Thoracotomy for the implantation of brain tissue extracts into the rat LV myocardium

The left thoracotomy protocol was described in detail in our previous report [43]. Briefly, during the preparation of the brain extract treatment, the animal was anesthetized by inhalation of 2.0% isoflurane and placed in the supine position on a warming pad at 37°C. Under sterile conditions, the heart was exposed via a left thoracotomy. The brain extract (60 μL/rat) was injected into the recipient heart at four points along the mid-portion of the anterior LV myocardium. The muscle layer and skin were carefully closed. Rats receiving thoracotomy only served as controls (NC group).

### *In vitro* study to determine the therapeutic effect of melatonin against DAMP inflammatory signaling

To evaluate the effects of melatonin on BD-tissue-induced DAMP inflammatory signaling, we used the following six groups of H9C2 cells: (1) normal control (NC; cells co-cultured with the reagent), (2) sham control (SC; cells co-cultured with healthy brain tissue extracts [1.0 μg/mL] for 24 hours), (3) BD_EX_ (cells co-cultured with BD_EX_ [1.0 μg/mL] for 24 hours), (4) BD_EX_-Mel (cells co-cultured with BD_EX_ and Mel [100 μM] for 24 hours), (5) BD_EX_-Mel-Luz^1^ (cells co-cultured with BD_EX,_ Mel [100 μM] and Luz [10 μM] for 24 hours) and (6) BD_EX_-Mel-Luz^2^ (cells co-cultured with BD_EX,_ Mel [100 μM] and Luz [20 μM] for 24 hours).

### Animal groupings for signaling pathway determination

To elucidate the endogenous DAMPs engaged with target receptors that induced the release of inflammatory mediators, we obtained 12-week-old adult male Sprague-Dawley rats (*n* = 12) weighing 320-350 g (Charles River Technology, BioLASCO Taiwan Co. Ltd., Taiwan) and divided them into the NC (*n* = 6) and BD (*n* = 6) groups.

To clarify the TLR2/4-NF-κB signaling pathway in the brain parenchyma of BD animals, we divided an additional 16 adult male Sprague-Dawley rats equally into the following four groups: NC, BD, BD + Mel (regimen described above) and BD + Mel + Luz (regimens described above).

### Animal grouping for the experimental study to test the protective effect of melatonin against BD-induced remote cardiac damage

To assess the protective effect of melatonin against BD-induced myocardial injury, we divided adult male Sprague-Dawley rats (*n* = 30) equally among the following five groups: (1) NC (only left thoracotomy), (2) SC (healthy brain tissue extracts [60 μL] implanted into the LV myocardium), (3) BD_EX_ (BD_EX_ [60 μL] implanted into the LV myocardium), (4) BD_EX_-Mel (BD_EX_ [60 μL] implanted into the LV myocardium, with the melatonin regimen described above) and (5) BD_EX_-Mel-Luz (BD_EX_ [60 μL] implanted into the LV myocardium, with the melatonin and luzindole regimens described above).

### Functional assessment by echocardiography

The method of transthoracic echocardiographic examination was standardized according to our previous reports [43]. Briefly, on days 0 and 5 after BD-derived tissue implantation, transthoracic echocardiography (Vevo 2100, Visualsonics, Toronto, Ontario, Canada) was performed by a veterinary cardiologist blinded to the experimental design. M-*mode* standard *two*-*dimensional* left parasternal *long axis* echocardiographic examination was conducted. The LV internal dimensions (LVESd and LVEDd) were measured at the mitral valve and papillary levels of the left ventricle, as per the American Society of Echocardiography (Morrisville, NC) leading-edge methodology, for at least three consecutive cardiac cycles. The LVEF was calculated as follows: LVEF (%) = [(LVEDd^3^-LVESd^3^)/LVEDd^3^] × 100%.

### Immunohistochemical and immunofluorescent staining

The immunofluorescent staining protocol was described in detail in our previous reports [31, 43, 44]. For immunohistochemical and immunofluorescent staining, rehydrated paraffin sections were treated with 3% H_2_O_2_ for 30 minutes and then incubated with Immuno-Block reagent (BioSB) for 30 minutes at room temperature. Sections were subsequently incubated with primary antibodies against CD14 (1:300, Bioss), CD68 (1:100, Abcam), CD3 (1:400, Abcam), CD4 (1:200, Novus Biologicals) and γ-H2AX (1:500, Abcam). Sections incubated with irrelevant antibodies served as controls. Three heart specimen sections from each rat were analyzed. For quantification, three randomly selected high-power fields (HPFs; 200× or 400× for immunohistochemical and immunofluorescent studies) were analyzed in each section. The mean number of positively-stained cells per HPF for each animal was then determined as the sum of all the numbers divided by 9.

### Western blot analysis

The Western blotting procedures were based on those in our recent reports [31, 43, 44]. In detail, equal amounts (50 µg) of protein extracts were loaded and separated by sodium dodecyl sulfate polyacrylamide gel electrophoresis with acrylamide gradients. After electrophoresis, the separated proteins were transferred electrophoretically to a polyvinylidene difluoride membrane (Amersham Biosciences). Nonspecific sites were blocked by incubation of the membrane in blocking buffer (5% nonfat dry milk in Tris-buffered saline containing 0.05% Tween 20) overnight. The membranes were incubated with the indicated primary antibodies for 1 hour at room temperature: cleaved PARP (1:1000, Cell Signaling), cleaved caspase 3 (1:1000, Cell Signaling), mitochondrial BAX (1:1000, Abcam), P53 (1:1000, Cell Signaling), MTR1 (1:200, Alomone Labs), MTR2 (1:200, Alomone Labs), SIRT1 (1:1000, Abcam), SIRT3 (1:500, Abcam), p-SMAD3 (1:1000, Cell Signaling), transforming growth factor ß (1:500, Abcam), TNF-α (1:1000, Cell Signaling), NF-κB (1:600, Abcam), IL-1β (1:1000, Cell Signaling), IL-6 (1:750, Abcam), TLR-2 (1:1000, Abcam), TLR-4 (1:1000, Abcam), HMGB-1 (1:1000, Cell Signaling), IL-10 (1:1000, Abcam), IL-4 (1:500, Abcam), cytosolic cytochrome C (1:1000, BD), mitochondrial cytochrome C (1:1000, BD), NOX-1 (1:1500, Sigma), NOX-2 (1:750, Sigma), γ-H2AX (1:1000, Cell Signaling), matrix metalloproteinase (MMP)-9 (1:2000, Abcam), IκB (1:1000, Santa Cruz), p-IκB (1:800, Santa Cruz), MYD88 (1:1000, Abcam), inducible nitric oxide synthase (iNOS) (1:1000, Abcam), IFN-γ (1:1000, Abcam) and actin (1:10000, Millipore). Horseradish peroxidase-conjugated anti-rabbit immunoglobulin IgG (1:2000, Cell Signaling) was used as a secondary antibody for 1-hour incubation at room temperature. The washing procedure was repeated eight times within 1 hour. Immunoreactive bands were visualized by enhanced chemiluminescence (Amersham Biosciences) and exposed to Biomax L film (Kodak). For the purpose of quantification, the enhanced chemiluminescent signals were digitized with Labwork software (UVP).

### Oxidative stress reaction in the LV myocardium

The protocol for assessing oxidative stress protein expression was detailed in our previous reports [31, 43, 44]. The Oxyblot Oxidized Protein Detection Kit was purchased from Chemicon (S7150). DNPH (2,4-dinitrophenylhydrazine) derivatization was carried out on 6 μg of protein for 15 minutes according to the manufacturer’s instructions. One-dimensional electrophoresis was performed on a 12% sodium dodecyl sulfate polyacrylamide gel after DNPH derivatization. Proteins were transferred to nitrocellulose membranes, which were then incubated in the primary antibody solution (anti-DNP, 1:150) for 2 hours, followed by incubation in the secondary antibody solution (1:300) for 1 hour at room temperature. The washing procedure was repeated eight times within 40 minutes. Immunoreactive bands were visualized by enhanced chemiluminescence (Amersham Biosciences) and exposed to Biomax L film (Kodak). For quantification, the enhanced chemiluminescent signals were digitized with Labwork software (UVP). For Oxyblot protein analysis, a standard control was loaded into each gel.

### Histological quantification of myocardial fibrosis and condensed collagen deposition

The protocols for histochemical studies of fibrosis, collagen deposition and infarct areas have been described previously [30, 43]. Masson’s trichrome and Sirius red staining were used to study fibrosis and collagen deposition in the LV myocardium, while hematoxylin and eosin staining was used to identify the infarct area. Three 4-µm-thick serial sections of the LV myocardium were prepared with a Cryostat (Leica CM3050S). The integrated area (µm^2^) of fibrosis, infarction or collagen deposition in each section was calculated with Image J software (National Institutes of Health). Three sections were quantified for each animal, and three randomly selected HPFs (100×) were analyzed in each section. The number of pixels in each diseased area per HPF was determined, and the numbers from the three HPFs were summed. The mean pixel number per HPF for each animal was then determined as the sum of all the pixel numbers divided by 9. The mean integrated area (µm^2^) of fibrosis, infarction or collagen deposition in the LV myocardium per HPF was then obtained.

### Statistical analysis

Quantitative data are expressed as means ± standard deviations. Statistical analysis was performed by analysis of variance followed by the Bonferroni multiple-comparison post hoc test. SAS statistical software for Windows version 8.2 (SAS Institute, Cary, NC, USA) was used. A probability value < 0.05 was considered statistically significant.
